# High glucose promotes hepatic fibrosis via miR-32/MTA3-mediated epithelial-to-mesenchymal transition

**DOI:** 10.3892/mmr.2022.12827

**Published:** 2022-08-16

**Authors:** Qiang Li, Zhange Li, Yuan Lin, Hui Che, Yingying Hu, Xujuan Kang, Ying Zhang, Lihong Wang, Yong Zhang

Mol Med Rep 19: 3190–3200, 2019; DOI: 10.3892/mmr.2019.9986

Subsequently to the publication of this paper, the authors have realized that Fig. 2 was published containing some incorrectly assembled data panels. The E-cadherin control data panel in Fig. 3F was re-used in Fig. 2C; furthermore, the HG / Vimentin data panel in Fig. 4E was re-used in Fig. 2D.

The authors have re-examined their original data, and were able to identify that Fig. 2 contained the erroneously assembled data panels. The revised version of Fig. 2, showing the correct E-cadherin control data panel for Fig. 2C and the correct HG / Vimentin data panel for Fig. 2D, is shown below. It was also noted that the white rectangles were not explained in the figure legend; these represent an enlargement of the cells in the E-cad/vimentin panels, and the details are now included in the figure legend (shown in bold). Note that these errors did not significantly affect either the results or the conclusions reported in this paper, and all the authors agree to the publication of this corrigendum. Furthermore, the authors thank the Editor of *Molecular Medicine Reports* for allowing them the opportunity to publish this corrigendum, and apologize to the readership for any inconvenience caused.

## Figures and Tables

**Figure 2.**
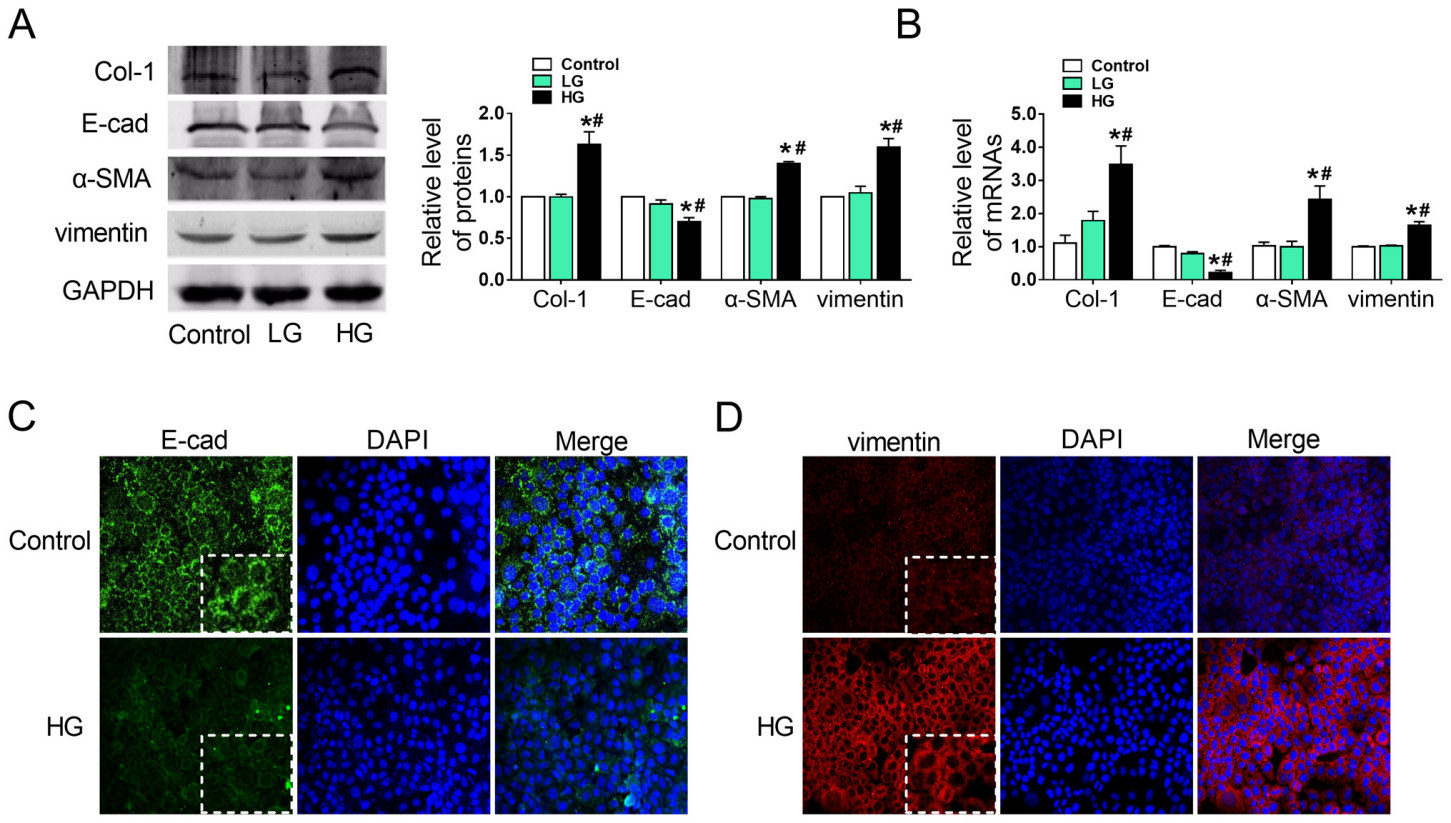
HG induces epithelial-mesenchymal transition in AML12 cells. (A) Alterations in Col-1, E-cad, α-SMA and vimentin protein expression in HG-treated AML12 cells was detected by western blotting, with representative blots on the left and relative quantification analysis on the right. (B) Relative mRNA expression of Col-1, E-cad, α-SMA and vimentin in HG-treated AML12 cells. GAPDH was used as an internal control. (C and D) Immunofluorescence images showing the location of EMT markers E-cad and vimentin in the control and HG-treated groups, with DAPI nuclear staining in blue, (C) E-cad in green and (D) vimentin in red (magnification, ×200). **The white rectangles represent enlarged cells within the same panel.** *P<0.05 vs. control; #P<0.05 vs. LG groups. E-cad, E-cadherin; α-SMA, α-smooth muscle actin; Col-1, collagen-1; HG, high glucose (6,000 mg/l); LG, low glucose (1,000 mg/l).

